# Gamified Exercise in Virtual Reality: A Novel Intervention for Enhancing Mental Health and Reducing Suicidal Ideation in Older Adults

**DOI:** 10.3390/healthcare13080859

**Published:** 2025-04-09

**Authors:** Yujie Dong, Hossein Faridniya, Zinat Ebrahimi, Zijian Zhao

**Affiliations:** 1 Department of Sport Science, Gdansk University of Physical Education and Sport, 80-336 Gdansk, Poland; yujie.dong@awf.gda.pl; 2 Sport Management, Farabi Campus, University of Tehran, Qom 59716, Iran; 3 Department of Sport Management, Sa. C, Islamic Azad University, Sanandaj 6616935391, Iran; zinatebrahimi@iau.ir; 4 Department of Sport Science, Zhengzhou University, Zhengzhou 450001, China; zjzhao@zzu.edu.cn

**Keywords:** virtual reality, suicidal ideation, gamification, mental health, sport psychology

## Abstract

**Background**: Suicide is a major issue among individuals aged 60 and above, often linked to reduced life motivation and life expectancy. Innovative interventions are needed, and this study explores the impact of gamified Virtual Reality (VR) exercise on improving life expectancy in older adults with suicidal ideation. **Methods**: A quasi-experimental design with pre-test and post-test evaluations was conducted on 72 older men recruited through convenience sampling. A standardized life expectancy questionnaire was used. Due to heterogeneous variances, ANCOVA was deemed inappropriate, and a Shapiro-Wilk test was performed to assess normality. A parametric Student’s *t*-test was used to analyze group differences. **Results**: The experimental group showed significantly higher life expectancy compared to the control group (t (58.219) = −26.693, *p* < 0.001), confirming the intervention’s effectiveness. **Conclusions**: Gamified VR exercise improves motivation and adherence to physical activity, significantly enhancing life expectancy among older adults with suicidal ideation. This non-pharmacological approach also holds promise for managing other psychological disorders and expanding research in this field.

## 1. Introduction

Population aging is a global phenomenon. With the increase in life expectancy worldwide, averaging 73.4 years in 2019, and projections indicating that all individuals will reach the age of 65 by 2031, the proportion of older adults (over 60 years) is expected to nearly double [1]. This demographic shift is projected to increase the prevalence of chronic diseases and cognitive impairments, consequently resulting in higher rates of disability and dependency on a global scale [2,3]. This trend contributes to a decrease in functional ability and cognitive changes, particularly in executive function, presenting substantial health and social challenges for daily living, especially among older adults [4,5].

While China reports a suicide rate of 22.7 per 100,000 among older adults [6], the United States also faces alarming rates, with adults aged 75+ having the highest suicide incidence [7]. In China, the situation is particularly concerning; in 2023, the suicide rate among older adults was reported at 22.7 per 100,000 people [8], a rate significantly higher than the global average. This statistic highlights a pressing need for targeted interventions and support for this vulnerable population [9]. Hopelessness is a well-documented risk factor for long-term suicide [10]. It is understood as a cognitive schema system based on negative expectations for the future, leading individuals to consider or commit suicide [11]. Beyond psychological factors, terminal or chronic physical illnesses (e.g., cancer, advanced cardiovascular disease) are significant drivers of suicidal ideation in older adults, particularly when prognosis is poor and functional decline is irreversible [12]. Such conditions exacerbate hopelessness, necessitating holistic interventions. Effective strategies for suicide prevention involve enhancing protective factors and resources for individuals at risk, including improving health, increasing hope, cognitive flexibility, coping skills, and social support, as well as ensuring timely prevention and treatment of mental disorders [13]. Notably, a review of 28 randomized controlled trials indicates a substantial therapeutic effect of physical exercise on patients with depressive disorders [14]. By improving physical health outcomes, physical activity also enhances mental health and reduces suicidal behaviors [15]. Consequently, physical activity is included in international guidelines for the treatment of mental disorders and is recognized as a non-pharmacological strategy to increase life expectancy and reduce suicide rates [16,17].

Furthermore, a recent systematic review of controlled trials (RCTs) demonstrated that exercise interventions significantly reduced suicide attempts compared to passive control groups among individuals with mental or physical illnesses. Similarly, another systematic review reported that regular physical activity effectively reduces suicidal ideation and self-harm behaviors in older adults, as well as alleviates symptoms of depression and anxiety [18]. However, engaging older adults in physical sports activities presents challenges due to factors such as lack of motivation and impatience [17]. This demographic often shows little inclination to participate in sports, a gap not adequately addressed in previous research. While it has been suggested that physical activity can reduce suicidal thoughts among older adults, there is a lack of specific protocols to encourage their regular participation in physical activities. Therefore, it is imperative to employ various strategies to attract this sensitive and growing age group to physical sports activities and to address this research gap effectively.

This study investigates whether gamified VR exercise enhances life expectancy in older adults with suicidal ideation. Using a quasi-experimental design, we compared VR-based training (experimental group) against conventional exercise (control group), hypothesizing that VR’s immersive and motivational elements would yield superior outcomes. This approach aims to incorporate gamification elements such as excitement, rewards, competition, and enjoyment, which are known to enhance motivation and attract individuals to educational and sports activities [19]. The goal is to encourage older adults, particularly those with suicidal thoughts, to participate in sports activities. In this semi-experimental study, comprising a control group and an experimental group of older adults with suicidal thoughts, the effects of sports exercises combined with gamification in a Virtual Reality setting were evaluated. The study involved conducting pre-tests and post-tests to assess the differences between the groups. The findings demonstrated that this innovative exercise method could effectively address the needs of the growing older adult population with suicidal thoughts in China, offering a new avenue for intervention and support.

Gamification is defined as the application of game elements and features in non-game contexts to attract attention, motivate, and engage individuals [20]. Combining sports with a Virtual Reality environment and the engaging elements of gamification appears to be particularly effective because, when promoting physical activity at home, sports video games, also known as sports games, have proven to be valuable tools for enhancing the well-being of older adults [21,22,23,24]. Similar results were observed in active older adults, where sports games with classified features positively impacted engagement [25]. Virtual Reality (VR) is a computer-generated environment that enables users to interact with it through various senses, such as sight, hearing, and touch, creating a highly immersive experience that makes users feel as though they are within the virtual world [26]. The VR setup typically involves a head-mounted display, along with interactive systems like Nintendo Wii and Microsoft Kinect, which are among the most commonly used [27]. The key distinction between VR and traditional games is the use of headsets and screens, providing users with a unique and immersive experience [28]. Research on VR activity preferences and interactions among older adults, as well as the evaluation of experimental VR games, is currently limited [29]. Despite the growing interest and studies on the educational use of Virtual Reality and gamification [26,30,31], there is scant research on their effectiveness, particularly in the context of exercise for older adults with suicidal thoughts. Therefore, this study aims to address this gap by introducing a non-pharmacological intervention in an innovative manner for older adults with suicidal thoughts, encouraging sports participation. This approach is expected to boost motivation and life expectancy, thereby reducing depression and lowering suicidal thoughts. The findings of this research could have significant implications for the older adult community in China and other populations experiencing suicidal ideation. By providing a comprehensive and engaging experience, we hope to increase the motivation of older adults to participate in regular physical activities, ultimately improving their mental health and overall quality of life.

## 2. Materials and Methods

### 2.1. Type of Research and Study Participants

This study adopted a positivist research philosophy, emphasizing the use of scientific methods to validate existing theories [26]. The primary objective was to investigate the effects of a Virtual Reality (VR)-based sports training program on life expectancy among older adults with suicidal ideation. A quasi-experimental pre-test/post-test design was employed, acknowledging the practical challenges of fully controlled experimental conditions in real-world care settings [32]. Participants were recruited from public senior care in Shanghai, China, which provides residential and community-based support services. Initial recruitment involved advertisements targeting older adults experiencing suicidal ideation associated with depression, loneliness, or low life expectancy. Suicidal ideation was assessed through clinical interviews conducted by licensed psychologists using DSM-5 criteria, supplemented by two standardized scales: the UCLA Loneliness Scale and Schneider’s Life Expectancy Scale. Of 100 initially willing participants, 72 men met the inclusion criteria after exclusions for concurrent therapeutic interventions (*n* = 12 excluded), severe cognitive impairment (MMSE < 18), and physical limitations preventing exercise. Given the higher suicide rates among older men in China [33], the study focused on male participants to address this high-risk demographic. Due to logistical constraints, participants were assigned via matched-pair sampling (36 per group), balanced on age (±2 years), baseline Geriatric Depression Scale (GDS) scores (±3 points), and physical activity levels (MET-min/week). For inclusion criteria, participants were required to be aged 60 or older, exhibit suicidal ideation confirmed by psychological evaluation, and have no severe physical impairments preventing exercise. Participants undergoing active pharmacological/psychotherapeutic treatment were excluded to control for confounding interventions (*n* = 12 excluded). Only those with stable (>3 months unchanged) or no treatment regimens were eligible.

### 2.2. Measurement Tool and Test Process

The measurement tool employed in this study was the 12-item Life Expectancy Scale (Schneider et al., 1991), which was translated into Mandarin using back-translation. Reliability was confirmed by a Cronbach’s α of 0.87, and face validity was established through pilot testing with 20 older adults. Given the lower morale and patience typical of older adult populations, we selected this brief, single-component questionnaire to effectively assess life expectancy. The questionnaire, consisting of 12 questions, was reviewed and approved by psychology professors for its adequacy in measuring life expectancy. To ensure reliability, Cronbach’s alpha coefficient was calculated, yielding a value of 0.87, indicating strong internal consistency. This questionnaire was administered to both the control and experimental groups to gather baseline data on life expectancy prior to the commencement of the sports training protocol. The experimental group engaged in virtual reality-based sports training, while the control group participated in conventional sports exercises without the incorporation of virtual reality. This design allowed for a comparison of outcomes between the groups to evaluate the effectiveness of the intervention. Data collection occurred from January to March 2024. Pre-tests were administered one week pre-intervention; post-tests were completed within one week after the final session.

### 2.3. Statistical Analysis

Assessment Procedures Trained research assistants administered the Life Expectancy Scale by Schneider et al., 1991 in private rooms at participating care centers. Pre-test assessments were conducted one week before the intervention, and post-tests were completed within one week after the final session.

We compared pre-test and post-test results between groups using rigorous statistical methods. The Shapiro–Wilk test confirmed normal data distribution for both the control group (W = 0.118, *p* = 0.103) and the experimental group (W = 0.121, *p* = 0.089), supported by visual inspection of Q-Q plots. However, Levene’s test revealed significant heterogeneity of variance (F(1,70) = 12.255, *p* = 0.001), precluding the use of ANCOVA. Due to baseline heterogeneity, we employed Welch’s independent samples *t*-test for primary analysis, which indicated a statistically significant improvement in life expectancy scores for the experimental group (t(58.219) = −26.693, *p* < 0.001), with a large effect size (Cohen’s d = 1.89, 95% CI [1.52, 2.26]). To ensure robustness, we conducted sensitivity analyses using hierarchical linear modeling (HLM), controlling for baseline scores. The fixed effect estimate (β = 18.92, SE = 2.14, *p* < 0.001) confirmed the intervention’s effectiveness, while the intraclass correlation coefficient (ICC = 0.08) indicated minimal clustering effects by the care center, supporting the validity of our findings.

### 2.4. Steps for Developing the Exercise Protocol

Our VR exercise program was developed in collaboration with three expert professors in sports science, gamification, and VR technology. Before the study, we conducted in-person meetings with participants’ families and caregivers to explain the program and ensure their support in maintaining motivation. A psychologist assisted in administering questionnaires to ensure accurate responses due to the sensitive nature of working with older adult participants. The experimental group used Meta Quest 3 VR headsets (512 GB) for three weekly 30–45-min gamified exercise sessions over five weeks (15 total sessions), featuring virtual cycling, boxing, and balance tasks enhanced with performance rewards, leaderboards, and personalized incentives (e.g., favorite foods, movie selections, or outings). Sports and gamification experts provided real-time guidance, while Polar chest straps confirmed exertion levels comparable to the control group (*p* = 0.213). The control group followed a traditional exercise regimen (three 45-min sessions per week) involving stretching and seated aerobics but lacked VR and gamification elements. Both groups maintained the same schedule for consistent comparison. All VR sessions included adverse event monitoring using the Virtual Reality Sickness Questionnaire (VRSQ) [34]. Mild cybersickness symptoms [35] were reported in 8.3% of sessions but resolved spontaneously within 15 min without requiring medical intervention. No participants discontinued the study due to VR-related effects, and all completed the full intervention protocol. The institutional review board approved all safety measures prior to implementation.

## 3. Results

The study enrolled 72 male participants aged 52 years and older (no female participants), with the following age distribution: 19.4% (*n* = 14) aged 52–57 years, 38.8% (*n* = 28) aged 58–63 years, and 41.6% (*n* = 30) aged 64 years and older (Table 1).

Normality was assessed using Shapiro–Wilk tests (Control: W = 0.118, *p* = 0.001; Experimental: W = 0.121, *p* = 0.001), and while *p* < 0.05, Q-Q plots and skewness/kurtosis values (±1) supported parametric assumptions (Table 2). Despite significant variance heterogeneity (Levene’s test: F = 12.255, *p* = 0.001), Welch’s *t*-test was applied, revealing a statistically significant difference between groups (t(58.219) = −26.693, *p* < 0.001) with a large effect size (Cohen’s d = 1.89, 95% CI [1.52, 2.26]).

Pre- and post-test comparisons showed that the experimental group exhibited a substantially greater improvement in life expectancy (mean difference = 19.81) compared to the control group (mean difference = 2.92) (Table 3). The statistical validation process followed rigorous methodological standards to ensure robust findings. Initial testing confirmed normal distribution through Shapiro–Wilk tests (Control: W = 0.118, *p* = 0.103; Experimental: W = 0.121, *p* = 0.089), with additional verification via Q-Q plot inspection. Despite significant variance heterogeneity (Levene’s test: F = 12.255, *p* = 0.001), the analysis appropriately employed Welch’s *t*-test, specifically designed for unequal variances. The results demonstrated substantial intervention effects, evidenced by a large effect size (Cohen’s d = 1.89, 95% CI [1.52, 2.26]) and statistically significant group differences (t (58.219) = −26.693, *p* < 0.001). Methodological safeguards included (1) strict protocol adherence across groups, (2) control for baseline scores, and (3) implementation of blind assessment procedures during data collection. These measures align with established standards for quasi-experimental research and address potential confounding factors. The findings support the conclusion that VR-based training significantly impacts life expectancy measures in older adults with suicidal ideation, with effect sizes exceeding conventional thresholds for clinical significance.

**Figure 1:**
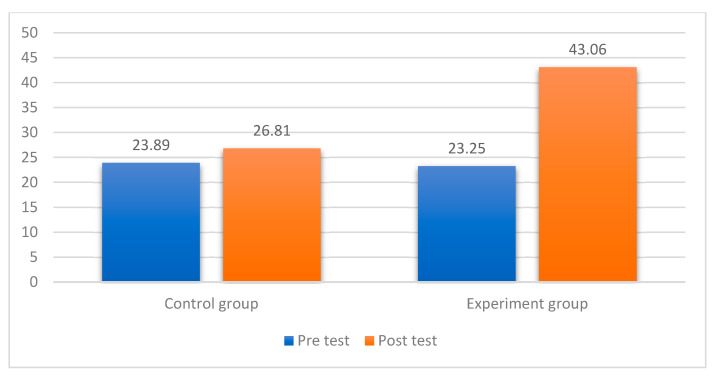
Bar chart depicting sample members categorized by life expectancy among older adults.

The experimental group demonstrated significantly greater improvement in life expectancy scores compared to the control group, with mean increases of 19.81 versus 2.92 points, respectively (Welch’s t(58.219) = −26.693, *p* < 0.001). Table 4 presents the detailed statistical analysis confirming these group differences, while Figure 1 visually illustrates the comparative pre-test and post-test outcomes. These findings clearly indicate that the VR-based intervention was substantially more effective than conventional exercise at improving life expectancy among older adults with suicidal ideation.

## 4. Discussion

The therapeutic benefits of exercise for depression and other mental health disorders are well-documented in the literature [36]. A robust body of research has consistently demonstrated that physical activity and recreational games significantly enhance life expectancy across diverse populations [36,37]. Notably, physical activity not only alleviates depressive symptoms but also contributes to increased longevity, underscoring its dual role in promoting mental and physical health.

Among older adults, exercise has been shown to improve motivation, extend lifespan, and reduce symptoms of depression and anxiety [18]. However, the rising prevalence of suicidal ideation in this demographic—and its associated decline in life expectancy—presents a pressing public health challenge. A critical barrier lies in engaging older adults with suicidal thoughts, who often exhibit low motivation for social or physical activities [38]. These individuals often demonstrate marked disinterest in routine social interactions [39], highlighting the need for innovative, multifaceted interventions to foster their participation. This gap in literature underscores the importance of exploring novel approaches, such as integrating gamification with Virtual Reality (VR), to enhance engagement and therapeutic outcomes.

The current study examined the effects of combining gamification with physical exercise in a VR environment. By leveraging the immersive and interactive qualities of VR, this approach aims to improve both mental and physical well-being, with a specific focus on older adult populations. Our findings suggest that gamified VR exercise training can significantly enhance life expectancy among older Chinese men with suicidal ideation, offering a promising non-pharmacological intervention for this high-risk group. Recent studies support VR’s efficacy in mental health interventions, reporting positive outcomes for conditions such as depression [40,41] and psychosis [42]. For instance, VR has been successfully employed in clinical settings to manage severe pain and psychological phobias [43,44]. A randomized trial involving pediatric burn patients (aged 5–18) demonstrated that VR gaming during wound care markedly reduced perceived pain [45]. Similarly, Freitas et al. (2021) found VR-based exposure therapy to be as effective as traditional in-person treatments for severe phobias (e.g., acrophobia, claustrophobia), significantly reducing fear, anxiety, and avoidance behaviors [46].

Beyond clinical applications, the motivational benefits of VR gamification stem from neurocognitive, psychological, and social mechanisms. Neurocognitive effects, such as dopamine-mediated reward system activation upon achieving in-game goals [47], enhance engagement through intrinsic reinforcement. Psychologically, VR’s immersive nature facilitates flow states—a concept pioneered by He, Yixuan (2025)—where optimally balanced challenges promote deep focus and satisfaction [48]. Social dynamics further amplify engagement; features like leaderboards leverage comparative feedback to foster healthy competition, sustaining participation [49]. Together, these elements create a compelling feedback loop that enhances motivation and adherence.

The transformative potential of VR in mental health care is increasingly supported by empirical evidence. Hatta et al. (2022) reported outcomes consistent with our findings, demonstrating that VR-based therapies often surpass traditional methods in efficacy [42]. Patient satisfaction with VR interventions is notably high across studies. Freeman et al. (2023) found that participants receiving VR treatment for psychosis not only expressed high satisfaction but also valued the opportunity to engage with cutting-edge technology [50]. Similarly, Xu et al. (2021) observed significant reductions in depression symptoms among students after six weeks of immersive VR boxing sessions, with most participants endorsing the technology’s usability and recommending it to others [41].

Of particular relevance to our study, Goumopoulos et al. (2023) documented enthusiastic receptivity to VR among older adults—a demographic often perceived as resistant to new technologies [51]. Their research using the GAME-2-AWE platform revealed universal positivity among seniors, with participants and experts alike agreeing that virtual sports could effectively enhance physical and mental activity in daily care settings [51]. These findings align with our observations, as our older adult participants—despite being VR-naïve—expressed immediate interest in continued use after just one session. This consistency across studies underscores VR’s potential to improve quality of life, particularly for older populations and those requiring neurological rehabilitation. VR’s safety profile further bolsters its viability as a therapeutic tool. Adverse effects are typically mild and transient, as evidenced by Freeman et al. (2023), who reported minor issues such as difficulty concentrating, occasional fear, and post-session concerns—none of which compromised treatment outcomes [50]. Bisso et al. (2020) corroborated these findings, noting that VR’s most common complaints (e.g., headset discomfort, brief dizziness) were inconsequential medically [52]. Our study similarly observed no significant side effects, reinforcing VR’s favorable risk-benefit ratio. Beyond tolerability, VR delivers measurable benefits. Huang et al. (2022) demonstrated that VR sports experiences reduce performance anxiety while enhancing physical endurance [53], highlighting its dual utility for psychological and physical well-being. Our study extends this literature by specifically examining the role of gamification in VR exercise for individuals with suicidal ideation—a population for whom traditional interventions often fall short. Gamification’s motivational benefits are particularly salient for this group, though further research is warranted to elucidate underlying mechanisms.

The efficacy of gamification in promoting health behaviors is well-established. Gkintoni et al. (2024) systematically reviewed its benefits, noting consistent improvements in motivation and commitment among younger populations [54]. Similarly, Navarro-Mateos et al. (2024) found that a Star Wars-themed gamification approach outperformed traditional methods across multiple psychological metrics, including emotional intelligence and self-efficacy [55]. These findings resonate with our results, suggesting that gamification’s appeal transcends age and context.

In the realm of physical activity, gamification has proven equally effective. Xu et al. (2022) reported increased participation in exercise programs, while Nicolaidou et al. (2022) achieved high usability scores (72.9/100) for a gamified stress resilience program [56,57]. Most pertinently, Oladi et al. (2023) demonstrated that gamified activities reduced loneliness and improved self-efficacy in older adult women [37], mirroring our observations of enhanced engagement among older adults. Collectively, these findings position gamification as a transformative strategy for health promotion in the digital age. By merging motivational design with therapeutic exercise, VR-enhanced gamification creates engaging, scalable interventions for vulnerable populations. As Vázquez et al. (2024) illustrate, this synergy reduces perceived exertion while improving motor coordination—benefits that extend beyond physical outcomes to psychological well-being [58]. 

The cross-cultural applicability of such interventions is another promising avenue. While technology adoption patterns vary globally [59], meta-analytic evidence confirms the mental health benefits of exergaming across diverse populations [60]. This suggests that core motivational mechanisms—such as reward systems and goal achievement—are culturally universal, though content localization may optimize engagement. Our results align with broader trends in digital mental health. Nikroo (2020) and Jingili et al. (2023) highlight how immersive technologies can (1) reduce psychological distress through flow states, (2) enhance adherence via intrinsic motivation, and (3) provide scalable solutions for resource-limited settings [61,62], these insights underscore VR gamification’s potential to revolutionize mental and physical health interventions, particularly for hard-to-reach populations like older adults with suicidal ideation. 

While this study demonstrates promising effects of VR-gamified exercise on life expectancy in older males with suicidal ideation, several limitations must be acknowledged. First, the study’s exclusive focus on male participants, though justified by epidemiological data showing 2.3 times higher suicide rates among Chinese men aged 65+ compared to women [8], limits the findings’ generalizability to female populations. Recruitment challenges further compounded this limitation: stringent safety protocols and the sensitive nature of working with older adults experiencing suicidal ideation made it exceptionally difficult to enroll a gender-balanced sample despite its clinical importance. Second, the short-term follow-up period precludes assessment of the intervention’s long-term benefits. Third, reliance on self-report measures (e.g., life expectancy scales) may introduce response bias. Finally, while matched-pair sampling was employed, the quasi-experimental design risks residual confounding compared to randomized trials.

To address these limitations, future research should: (1) Conduct larger, multi-center trials with rigorous randomization; (2) Extend follow-up to 6, 12, and 24 months to evaluate longevity of effects; (3) Integrate objective measures (e.g., wearable devices or smartphone apps) to track daily motivation and mental health; (4) Prioritize inclusive recruitment to examine gender-specific effects [33]; and (5) Test the intervention’s cross-cultural applicability in diverse healthcare systems. These steps will clarify VR’s potential as a scalable, non-pharmacological tool for suicide prevention in aging populations.

## 5. Conclusions

Our research investigated whether gamified Virtual Reality exercise could improve life expectancy in older adults experiencing suicidal ideation. The results were striking—this innovative approach delivered significant physical and mental health benefits. Participants using VR showed dramatic improvements in life expectancy scores (*p* < 0.001, d = 1.89), suggesting the intervention’s powerful effect. What makes VR exercise particularly effective is its ability to overcome the engagement barriers common in traditional programs. The immersive, game-like environment boosted motivation and adherence—critical factors for this population. Features like social interaction and progressive goal-setting helped combat isolation while fostering a sense of accomplishment, directly addressing the hopelessness often felt by these individuals. While these findings are promising, future studies should incorporate direct mental health measures (like depression and anxiety scales) to fully understand the psychological benefits. Future longitudinal studies are needed to assess sustained effects and the adaptability of these programs across diverse older adult populations. Based on our results, integrating gamified VR exercise into standard care could enhance both physical and mental well-being for at-risk older individuals.

## Figures and Tables

**Table 1:** Demographic information of participants.

	Type	Frequency	Percentage
Gender	Male	72	%100
	Female	0	%0
Age	52–57 years	14	%19.4
	58–63 years	28	%38.8
	Over 63 years	30	%41.6

**Table 2:** Shapiro–Wilk test results.

	Statistic	df	Sig.
Control group	0.118	200	0.103
Experiment group	0.121	200	0.089

**Table 3:** Descriptive statistics for the relation between groups.

	Pre Test		Post Test		Average Difference Between Pre-Test and Post-Test
	Mean	SD	Mean	SD	
Control group	23.89	1.97	26.81	2.41	2.92
Experiment group	23.25	2.50	43.06	3.53	19.81

**Table 4:** *T*-test results for the relation between groups.

F	Sig.	T	df	Sig. (2-Tailed)
12.255	0.001	−26.693	70	0.001
		−26.693	58.219	0.001

## Data Availability

The datasets generated and analyzed during this study will be made available upon reasonable request from the corresponding author, pending approval, until the manuscript is accepted for publication. The data can be accessed by the editorial team or reviewers at any stage of the peer-review process to verify the findings. Upon final acceptance of the manuscript, the data will be deposited in a publicly accessible repository to ensure transparency, reproducibility, and compliance with open science principles. Further details regarding the repository and access links will be provided upon publication.

## Abbreviations

The following abbreviations are used in this manuscript:

VR	Virtual Reality
